# Coke Deposition in CHA Zeolite Predicted by CO_2_ Adsorption Isotherms and Molecular Simulation

**DOI:** 10.1021/acsomega.5c06379

**Published:** 2025-10-23

**Authors:** Daniel V. Gonçalves, Beatriz O. Nascimento, Hilldyson M. Levy, Moises Bastos-Neto, Sebastião M. P. Lucena

**Affiliations:** † Laboratory of Modeling and 3D Visualization, Department of Chemical Engineering, 28121 Universidade Federal do Ceará, Campus do Pici, bl 709, 60455-760 Fortaleza, CE, Brazil; ‡ Laboratory of Adsorption and CO_2_ Capture, Department of Chemical Engineering, Universidade Federal do Ceará, Campus do Pici, bl 709, 60455-760 Fortaleza, CE, Brazil

## Abstract

Molecular sieves used in catalytic and adsorptive processes in the petroleum/petrochemical industry are subject to deactivation by coke deposition. Here, we apply CO_2_ adsorption isotherms associated with molecular simulation models to extract additional information from the coke deactivation process. We developed a predictive molecular simulation model for naturally occurring zeolite CHA with multiple cation mixtures. The model was validated based on experimental CO_2_ adsorption isotherms. *N*-heptane, benzene and naphthalene were tested as model coke molecules to describe deactivation as a function of carbon concentration. The *n*-heptane molecule was the one that best represented coke from the available experimental data. In addition to predicting deactivation, the combined use of CO_2_ adsorption isotherms with the molecular simulation model can discriminate the nature of coke molecules. The procedure described can be applied to other molecular sieve structures, in catalysis and adsorption, and to other model molecules for coke.

## Graphical abstract

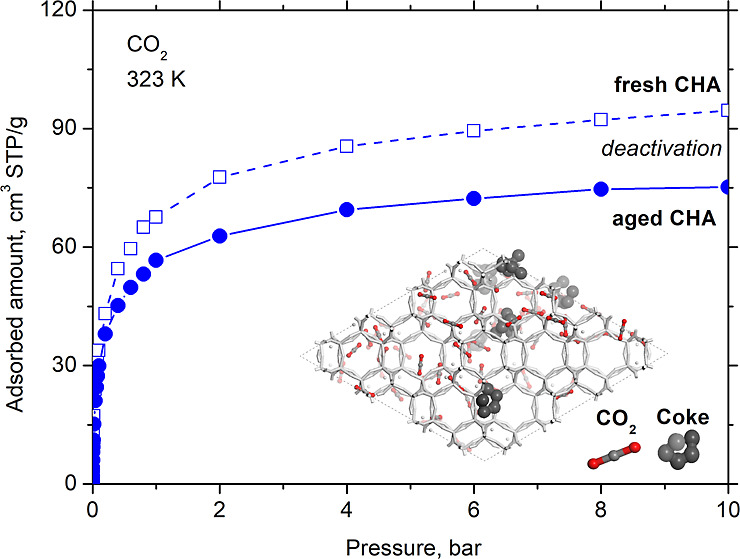

## 1 Introduction

Chabazite (CHA) is one of the most widely used natural zeolites in industry due to its small pores, hydrophilic framework and stability, which render it ideal properties for adsorptive drying of gases. The industrial use of adsorbents was made possible through the swing adsorption technique, with gas drying being one of the earliest applications.^1^ When employed in natural gas facilities, drying is the first unit operations, following gas extraction from the reservoir, which is performed usually by swing the temperature (Temperature Swing Adsorption, TSA) to continuously reuse the adsorbent. Under these conditions, drying molecular sieves are prone to progressive deactivation due to the presence of heavier hydrocarbons (C4+), even in trace amounts. Zeolite deactivation is well documented in the literature when this molecular sieve is used as catalyst,^2,3^ usually in the protonic form. However, the deactivation of cationic zeolites used in adsorption separation processes is scarce in the literature. The adsorption of heavy hydrocarbons in CHA has been addressed by Daems et al.^4^ and Cosseron et al.^5^ Daems et al.^4^ observed that, starting from C5, hydrocarbons experienced steric restrictions to diffuse through the windows giving access to pores, which reduces significantly its adsorption uptake. Cosseron et al.^5^ investigated the adsorption of *n*-hexane in zeolite structures STT, MFI, BEA and CHA. Although a rise in temperature to 150 °C reduced the adsorption capacity in STT, MFI and BEA, unlike other zeolites, the access of *n*-hexane was favored with the temperature rise, indicating less severe diffusion/steric resistances. In the complex mixture defined as natural gas, traces of heavy hydrocarbons may diffuse into the molecular sieve and, under temperatures typically required for water desorption (over 200 °C), polymerization leading to coke deposition may take place inside pores. Most studies to identify the chemical nature of coke and its impact on molecular sieve properties were carried out in the 1990s and early 2000s.^6^ Recent advances have employed multiple spectroscopic techniques to investigate the chemical nature, quantity, and location of coke molecules, and their impact on the methanol–olefin reaction in SAPO-34, which has the same topology as CHA.^7–9^ Gao et al.^7^ used 7–10 μm SAPO-34 single crystals to investigate in detail how the amount of coke impacted the surface area reduction, in addition to discussing its chemical nature. Wang et al.^8^ studied coke formation in detail and proposed a growth mechanism in which the aromatic coke molecule forms bonds through the windows connecting the different cavities. Zuo et al.^9^ proposed SAPO-34 zeolite precoke to increase ethene selectivity in the methanol-to-olefins process. According to the authors, the addition of some aromatic coke molecules reduces the diffusion of the reaction products, promoting an increase in selectivity. Studies with coke in other sieves have also been examined in more detail.^10^

In 2019, our group developed a lab-scale protocol to accelerate adsorbent (CHA) aging under the typical operating conditions in TSA drying of natural gas.^11^ CO_2_ adsorption isotherms at 323 K were measured to monitor the textural properties (micropore volume) of the material and a progressive reduction in CO_2_ uptake was observed for samples aged with increasing aging time. This aging protocol has also been recently applied to assess adsorbent deactivation with respect to adsorption equilibrium (N_2_ at 77 K, CO_2_ at 273 K and H_2_O at 303 K) and mass transfer kinetics for CHA and LTA (H_2_O at 303 K).^12^ These studies have demonstrated the relevance of CO_2_ as probe gas for the textural characterization of these materials with the progressive decrease in uptake due to coke formation and the increasing diffusional limitations, which ruled off N_2_ isotherms at 77 K as the assessment method of choice. On the other hand, even though analytical techniques (FTIR, CHN and TGA) indicated the presence of aromatic carbon deposited in aged LTA, only aliphatic carbon was identified in CHA.^12^ As a matter of fact, this experimental evidence corroborates the findings of Guisnet and Magnoux,^3^ who state that the nature of coke tends to be aromatic in zeolites with larger pores than in those with small to intermediate pore sizes. Note that the largest cage diameter in LTA is 11.05 Å, whereas it is only 7.37 Å for CHA. This is an excellent opportunity to apply molecular simulation tools to verify the possibility of representing coke with relatively simple model molecules as suggested by Guisnet and Magnoux^3^ using CO_2_ adsorption isotherms for validation and characterization. It is important to note that CO_2_ is normally not recommended for characterization of zeolites due to its strong interaction with the structure, however, in this case involving coke, CO_2_ plays a strategic role due to the rapid diffusion capacity^13^ considering that at small concentrations of coke it is no longer possible to use N_2_. Another convenience of using CO_2_ is that the measured decreases in adsorption as the coke concentration increases are proportional to the loss of drying capacity of the sieve.^12,14^

Molecular simulation has been applied in the study of coke formation in zeolites used as catalysts.^15,16^ Migliori et al.^15^ investigated the preferred site of deposition of three model coke molecules in the pores of MFI, MOR and FER sieves. Four model coke molecules of increasing molecular weight were investigated by Martin and Guisnet,^16^ where the objective was to verify how the preferred deposition sites in the pores of the H-MFI zeolite were affected as the chain size of the model coke molecules increased. To the best of our knowledge, our study is the first to investigate the impact of coke deposition using adsorption isotherms and to demonstrate how a molecular simulation model can predict the relationship between coke concentration and sieve deactivation. This novel framework provides an alternative to purely experimental methods, enabling the prediction of deactivation trends without the need for costly and time-consuming experimental testing. Furthermore, it delivers molecular-level insights into how structural modifications induced by coke formation lead to the progressive deactivation of the adsorbent.

We propose a CHA structure with mixed cations to represent the naturally occurring zeolite reported by Nascimento et al.^12^ Then, we developed and tuned a force field that is capable of modeling CO_2_ adsorption in the mixed cation CHA. We carried out the virtual aging of the CHA structure by inserting coke-model molecules in the framework to predict the zeolite deactivation observed experimentally. The transferability of the methodology to other zeolites (LTA and SAPO-34) and probe molecules were also studied.

## 2 Models and Methods

### 2.1 Models

#### 2.1.1 CHA-type Zeolite

The unit cell of chabazite was built based on the model proposed by Andersen et al.^17^ The structure has *a* space group of type R-3m, with a = b = 13.5799 Å, c = 14.7472 Å and ratio Si/Al = 3. The Löwenstein rule^18^ was applied to replace Si atoms for Al, to forbid Al–O–Al bonds.

To conveniently represent a naturally occurring CHA, three types of cations (Ca, Na e K) were inserted in the structure (Figure 1), according to the chemical analysis reported in Santiago et al.,^11^ resulting in a simulation cell with framework composition of Ca_24_Na_12_K_12_Si_216_Al_72_O_576_. The cations were randomly distributed among the sites located in the eight-membered ring (8R) and six-membered ring (6R) windows. The 8R window has 4 possible positions called B sites, while the 6R windows have 3 possible positions called A’ (Figure 1). In our model approximately 90% of the cations occupy type B sites while 10% occupy type A’ sites. This distribution compares with that found in the study by Andersen et al.,^17^ where 80% of the cations occupied positions in the B site.

**Figure 1:**
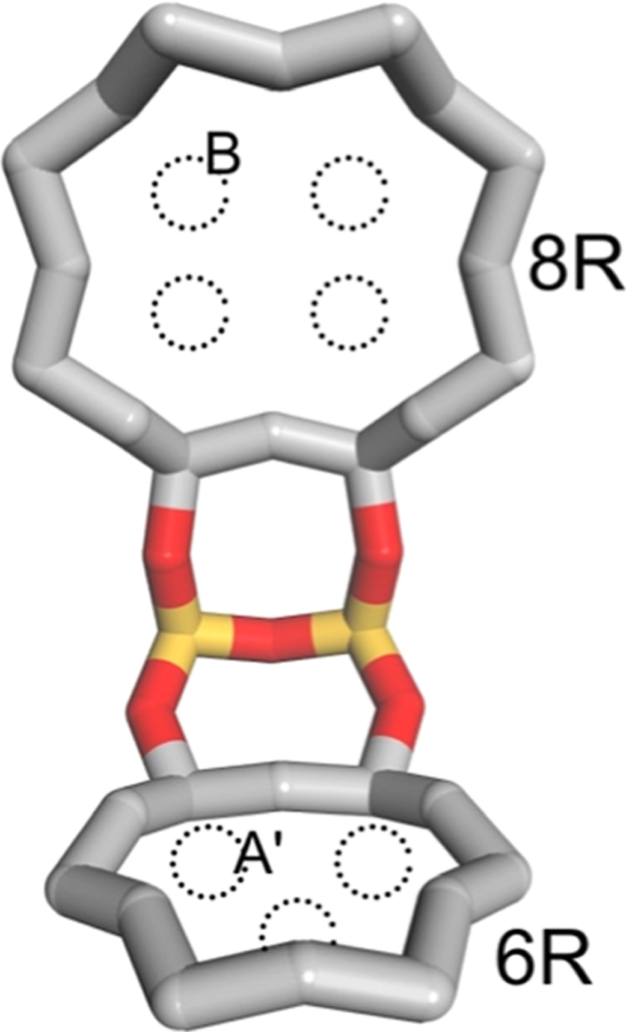
Positioning of the adsorption sites B and A’ in the 8R and 6R windows respectively, inside the CHA cage.

We know that zeolites with a Si/Al ratio other than 1, we must pay attention to the distribution of aluminum atoms in the structure because they interfere in the cation distribution. When studying the influence of the distribution of Al atoms on the structure of Na-LTA, Na-CHA and Na-FER zeolites, Findley et al.^19^ found that the CO_2_ isotherms of Na-CHA are consistent with random ordering of Al, which justifies our procedure of randomly distributing cations. Force field parameters based on UFF^20^ and atomic charges were obtained by the Charge Equilibration method (QEq),^21^ as summarized in Table 1.

**Table 1:** Force Field Parameters and Atomic Charges for CHA

atom	σ, Å	ε/k_B_, K	q, e^–^
Si	3.82	2.31	1.208
Al	4.01	2.92	1.2
O	3.12	35.22	–0.67925
Ca	3.03	119.79	1.22
Na	2.65	15.09	0.61
K	3.39	17.61	0.61

#### 2.1.2 Carbon Dioxide

The model EPM2 with three sites has been considered for CO_2_, as proposed by Harris and Yung.^22^ Three-site models of CO_2_ accurately account for liquid–vapor equilibria and have been successfully employed in the development of force fields for FAU^23^ and LTA^24^ zeolite structures, as well for activated carbons^25^ and MOF.^26,27^ Table 2 condenses the force–field parameters and atomic charges proposed by this model.

**Table 2:** Force Field Parameters and Atomic Charges for CO_2_

atom	σ, Å	ε/k_B_, K	q, e^–^
C	2.76	28.18	0.653
O	3.03	80.51	–0.3265

#### 2.1.3 Coke Molecules

In agreement with the experimental aging protocol previously reported^11^ and following the work of Guisnet and Magnoux,^3^ the molecules *n*-heptane, benzene, and naphthalene were selected to represent a spectrum of coke structures with increasing molecular size, complexity, and deactivation potential. Specifically, *n*-heptane was used to represent linear aliphatic coke formed in CHA, benzene to model monoaromatic species, and naphthalene to simulate polyaromatic coke. This tiered selection enables our model to evaluate the distinct deactivation effects caused by different coke natures. All these organic molecules were represented according to the TraPPE-UA model^28^ (Table 3).

**Table 3:** Force Field Parameters for Organic (Coke) Molecules

pseudoatom	σ, Å	ε/k_B_, K
C (naphthalene)	3.7	30.0
CH (benzene)	3.695	50.5
CH2	3.95	46.0
CH3	3.75	98.0

### 2.2 Virtual Aging Procedure

Coke molecules were inserted in CHA by means of configurational-bias Monte Carlo simulations in the canonic ensemble (NVT).^29^ This procedure allows that a given number of coke molecules is positioned in preferential sites of the zeolite framework. Simulations were performed with the RASPA 2.0 code.^30^ van der Waals interactions were calculated by the Lennard-Jones potential, and they were truncated in 12.8 Å. The Lorentz–Berthelot (LB) mixing rules were employed to calculate the solid–fluid interaction parameters. A periodic boundary condition was considered, and the CHA unit cell was replicated to meet the condition of minimal imaging. No tail correction was applied. 30,000 Monte Carlo cycles were carried out until the system came to a final configuration, which was taken as the aged chabazite sample.

### 2.3 Adsorption Isotherm

CO_2_ adsorption isotherms were calculated through Monte Carlo simulations applied to the Grand Canonical ensemble (GCMC). These calculations were also performed using the RASPA code 2.0.^30^ Again, we apply the periodic boundary condition and a cutoff radius of 12.8 Å. To satisfy the minimum image condition, the unit cell was duplicated in three dimensions. The truncated Lennard–Jones equation without tail correction was used to account for the van der Waals interactions. Solid–fluid parameters were obtained using LB mixing rules. The Ewald sum was used to compute the electrostatic interactions, with a cutoff distance of 12.8 Å and a precision of 10^–6^. 20,000 Monte Carlo cycles were used in the equilibrium phase and 40,000 cycles to account for the system averages. The conversion of the absolute adsorbed amount (resulting from the simulation) to the excess amount (experimentally measured) was performed using the Peng–Robinson equation.

## 3 Results and Discussion

### 3.1 Adsorption of CO_2_ in Fresh CHA

To validate the force field, we took the zeolite CHA simulation cell, described in the methodology, and simulated the CO_2_ adsorption isotherm at 323 K (Figure 2). This simulated isotherm, shown in Figure 2, can be compared with the experimental isotherm performed by Santiago et al.^11^ in virgin natural CHA (before the aging treatment). We observed that the simulated isotherm overestimates the experimental values, especially from 1 bar. Natural zeolites incorporate impurities that do not contribute to adsorption, typical of the natural genesis process, such as quartz, smectite and cristobalite,^31^ in addition to these impurities, the material was also pelleted. Based in typical impurities concentrations,^32^ we estimate that approximately 25% of its mass is composed of inert that do not contribute to adsorption. Our ideal crystal model does not account for the presence of impurities. At low pressures, the simulated and experimental isotherms are more similar because the most energetic adsorption sites are occupied first, and these sites in the natural zeolite are readily available. Consequently, the initial CO_2_ molecules adsorb without restriction. However, as the pores approaches saturation, the inert components present in the experimental sample begin to reduce the available adsorption volume, lowering the measured uptake in cm^3^ of CO_2_ per mass of adsorbent. The experimental isotherm reflects the amount adsorbed per total mass of the sample, whereas the simulation reports adsorption per total mass of pure CHA. This difference in sample composition is therefore the primary cause of the observed discrepancy between experimental and simulated isotherms. In addition, natural materials may exhibit structural defects or retain residual synthesis additives, which can contribute to minor deviations. Further discrepancies between experimental and simulated results may also arise from intrinsic limitations of both approaches, encompassing experimental factors (e.g., sample preparation and measurement uncertainties) and modeling assumptions (e.g., the rigid framework approximation and the imposed boundary conditions). We emphasize that the shape of the curves is similar, applying a linear reducer to the simulated curve, it practically coincides with the experimental one. This aspect is important because it demonstrates that the equilibrium of CO_2_ adsorption in the pores of our model represents the equilibrium in the real material. The agreement between the simulated and experimental isotherms indicates that the model and force field parameters that were developed for the CO_2_–CHA system are suitable for predicting CO_2_ adsorption.

**Figure 2:**
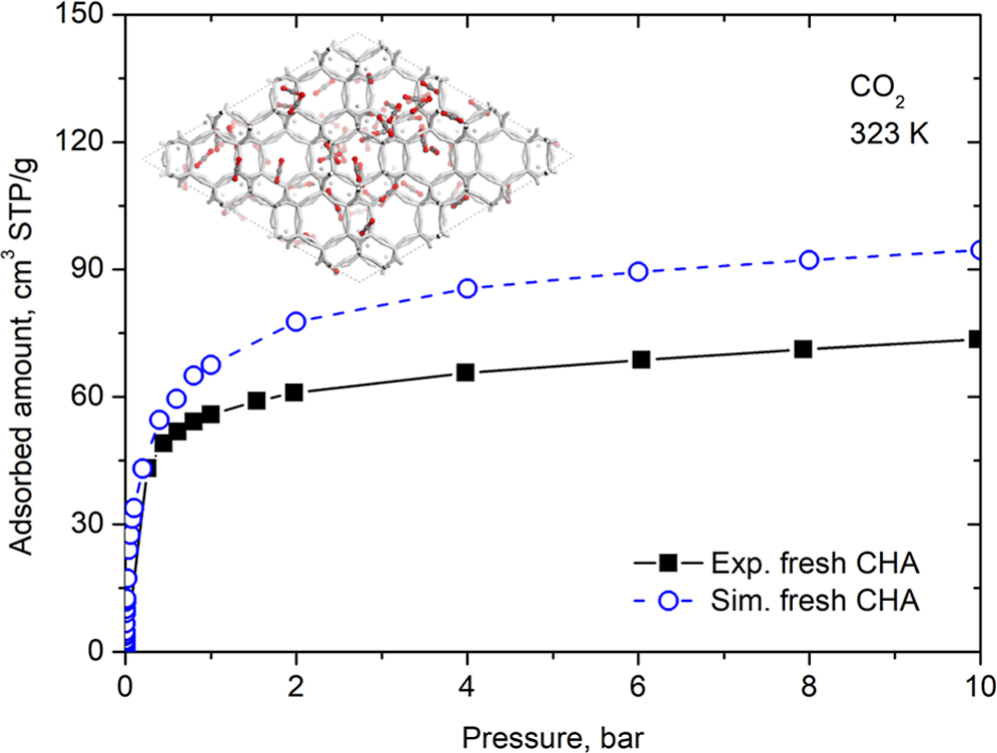
Adsorption isotherms of CO_2_ at 323 K in fresh CHA. Experimental data were taken from Santiago et al.^11^ A representative snapshot from the molecular simulations is shown to illustrate the adsorption configuration. CO_2_ molecules are displayed in gray (carbon atoms) and red (oxygen atoms).

### 3.2 Virtual Aging

Once the molecular model and force field parameters were validated, we used the NVT ensemble to generate virtually aged structures by inserting *n*-heptane molecules into the CHA. From 1 to 6 n-C7 molecules per simulation cell were introduced into the CHA. We obtained aged CHA structures with different degrees of coke concentration (% carbon). Figure 3 shows an example of a resulting structure with 6 molecules of *n*-heptane per unit cell which corresponds to 2.66% carbon in the structure. The *n*-heptane molecules occupy the CHA cages, one molecule per cage, like the positioning obtained in the study by Krishna and van Baten.^33^ This procedure was also performed to obtain structures with deposited benzene and naphthalene molecules to investigate the sensitivity of the model and the nature of the coke formed in the CHA. We acknowledge that this virtual aging procedure does not capture the detailed reaction kinetics of coke formation, such as high-temperature hydrocarbon polymerization. Nevertheless, it provides a practical and computationally efficient approximation of coke accumulation for investigating adsorption and deactivation.

**Figure 3:**
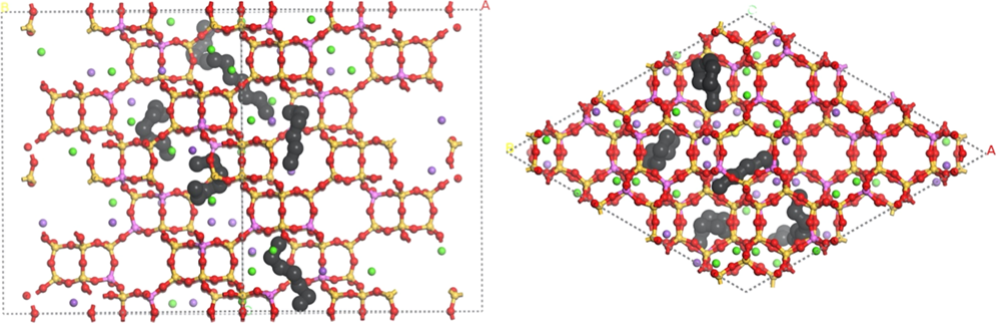
Snapshot of the CHA simulation cell with 6 molecules of *n*-heptane seen in planes 110 (side) and 001 (top). The colors dark gray, pink, yellow, and red represent C, Al, Si, and O atoms. Green are Ca^2+^ cations and purple are Na^+^ and K^+^.

### 3.3 Adsorption of CO_2_ in Aged CHA

In addition to virgin chabazite, Santiago et al.^11^ also measured the CO_2_ adsorption isotherm at 323 K in chabazite with different degrees of aging (% coke). We took the most aged sample for study. Elemental analysis of this sample indicated a carbon percentage of 2.35%. Thus, we used our virtually aged chabazite with 6 molecules of *n*-heptane (which corresponds to 2.66 wt % of carbon) and calculated the CO_2_ isotherm at 323 K. Figure 4 shows the comparison between the two isotherms. Once again, we observe a good correspondence between the experimental and simulated isotherms. The reasons for the observed discrepancy between the simulated and experimental isotherms were already discussed in Section 3.1.

**Figure 4:**
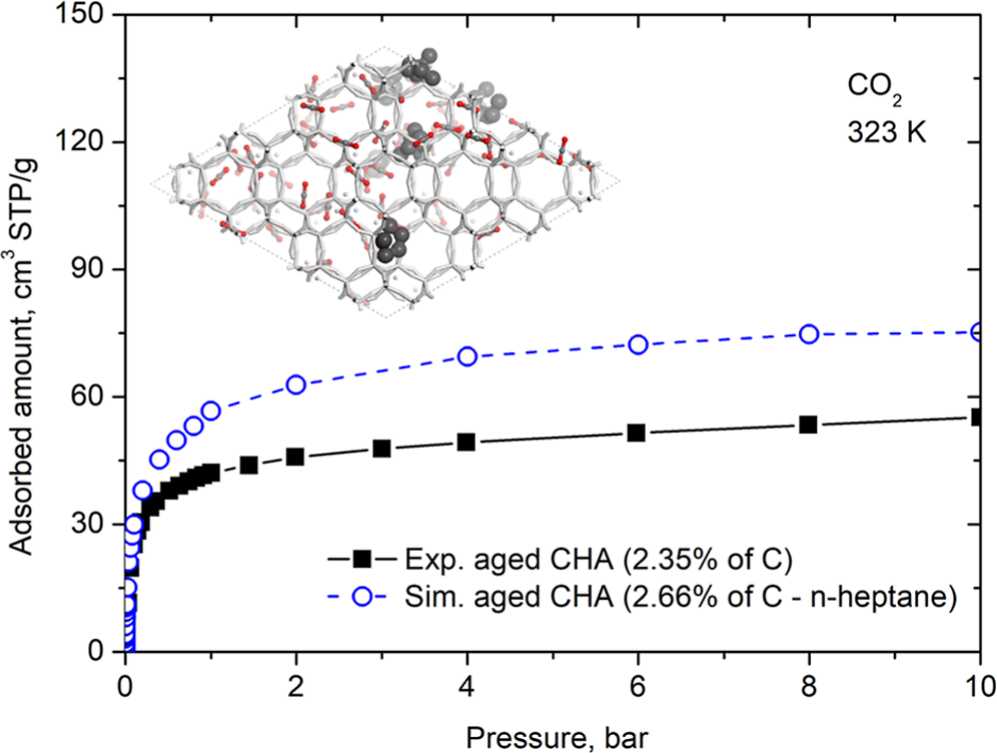
Adsorption isotherms of CO_2_ at 323 K in aged CHA. Experimental data were taken from Santiago et al.^11^ A representative snapshot from the molecular simulations is shown to illustrate the adsorption configuration. CO_2_ molecules are shown in gray (carbon atoms) and red (oxygen atoms), while *n*-heptane (coke) molecules are displayed in dark gray.

### 3.4 Deactivation of CHA

To quantitively assess the impact of carbon deposition, we define the metric “absolute deactivation” as the difference in adsorption capacity between the fresh and the aged (coke-loaded) zeolite under identical pressure and temperature conditions. From the adsorbed amounts calculated on virgin and aged CHA, we calculated the absolute deactivation defined as the difference between the amount of CO_2_ at 10 bar and 323 K in the virgin sample and the amount of CO_2_ in the samples aged under the same conditions. In addition to the aged CHA with 6 molecules of n-C7 per simulation cell, that was used in the previous section, here we also use the structures with 1, 2, 3, 4, and 5 n-C7 to generate a graph of absolute deactivation as a function of percentage of carbon in the CHA (Figure 5). We compared the simulated values in the different coke concentrations with those obtained with the experimental isotherms measured in the four different samples of CHA aged with 0.978%, 1.643%, 2.032% and 2.354% of C, reported by Santiago et al.^11^ Our deactivation prediction from the simulated data almost coincides with the values of the experimental data. We also observed that, in this concentration range, there is a linear trend that relates the percentage of C and the deactivation of CHA. After interpolation to the experimental coke loadings, the predicted absolute deactivation shows a Mean Absolute Percentage Error (MAPE) of 11.6% across all samples. The agreement is notably strong at higher coke concentrations (>1.5%), where the model achieves a MAPE of only 2.59%. This result is evidence that, with molecular simulation techniques, we can predict the deactivation of different CHA structures based on the carbon mass of the sample. As we mentioned earlier, we know that the deactivation measured with CO_2_ isotherms is closely related to the deactivation of the drying capacity of the sieve.^12^

**Figure 5:**
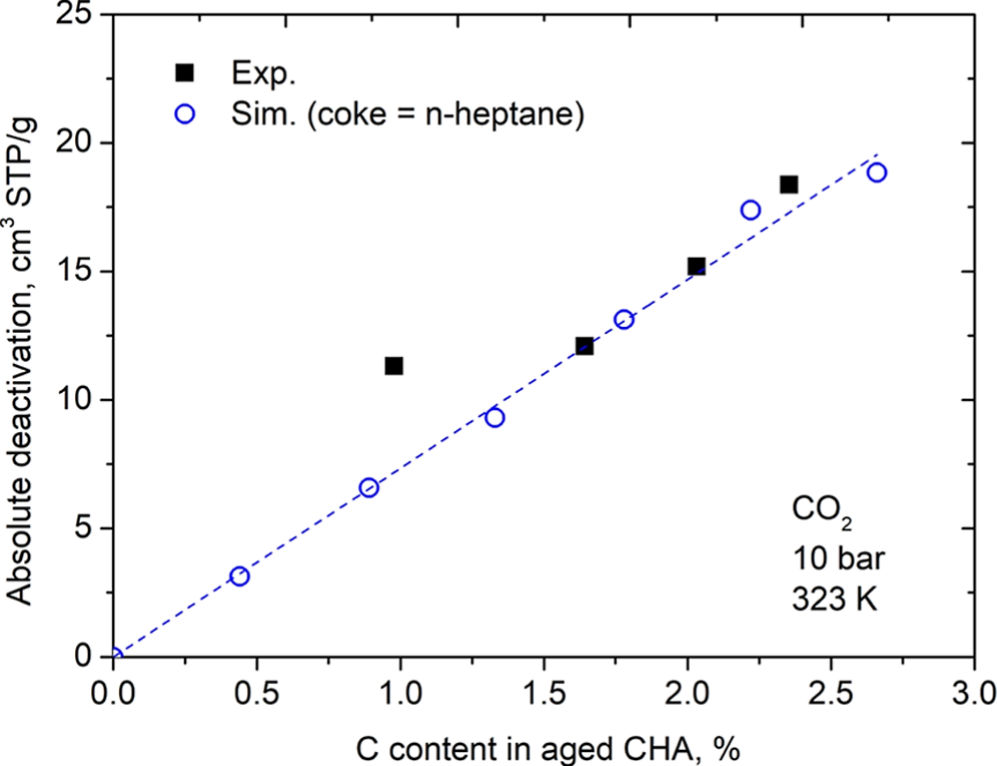
Absolute deactivation of CO_2_ at 323 K and 10 bar versus C content in aged CHA. *n*-Heptane was used as coke-model in theoretical calculations. Data taken from Santiago et al.^11^ were used to carried out experimental calculations. The dashed line is provided as a visual guide (linear fit).

To gain deeper insight into the mechanistic aspects of deactivation, we quantified the structural changes induced by coke deposition by calculating the CO_2_-accessible pore volume for both fresh CHA and virtually aged CHA models containing *n*-heptane. These calculations were performed using the Connolly surface method^34^ with a probe radius of 1.65 Å, corresponding to the kinetic diameter of a CO_2_ molecule,^35^ and a grid interval of 0.25 Å. The loss of accessible volume was determined from the difference between the pore volume of fresh CHA and that of the aged structures. As shown in Figure 6, a nearly linear correlation is observed between the accumulated *n*-heptane load and the percentage loss of accessible pore volume. Regression analysis indicates that for every 1% increase in carbon content from *n*-heptane loading, approximately a 1.8% reduction in accessible pore volume occurs. This linear trend in pore volume loss is consistent with the linear behavior of CO_2_ deactivation presented in Figure 5, together suggesting that the primary deactivation mechanism for this linear aliphatic coke is steric pore blockage. The coke molecules physically occupy the internal volume of the chabazite cages, progressively hindering CO_2_ adsorption in portions of the porous network. This analysis directly links the macroscopic loss of adsorption capacity to microscopic structural alterations, thereby providing a more comprehensive understanding of the deactivation process.

**Figure 6:**
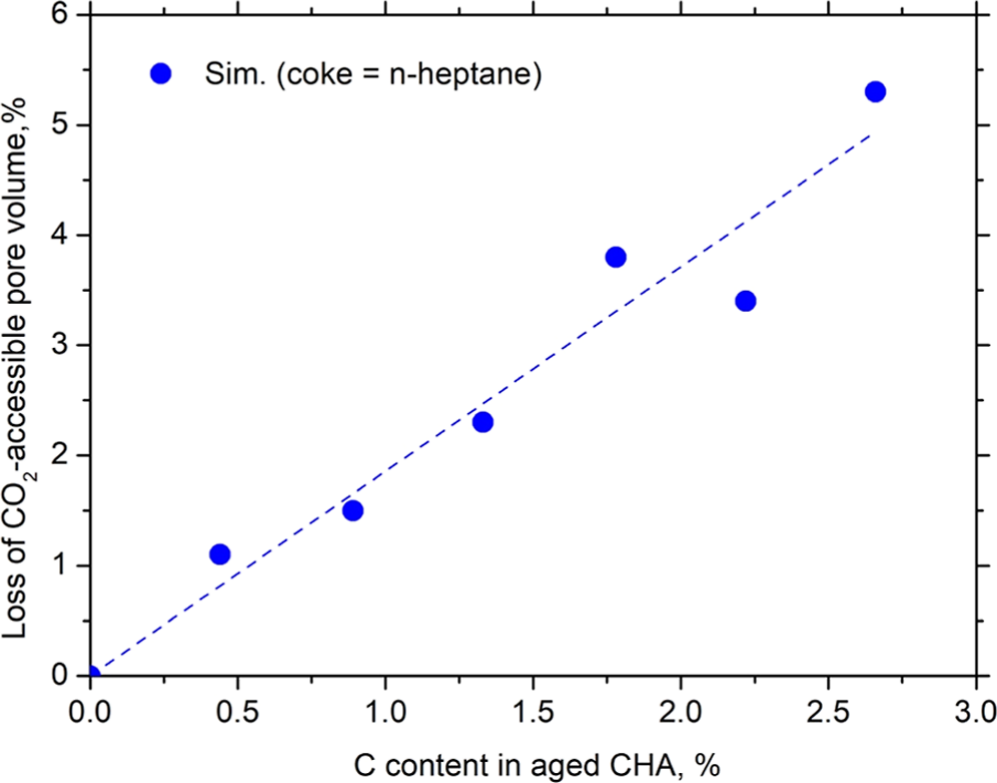
Correlation between C content in aged CHA (coke as *n*-heptane) and the loss of CO_2_-accessible pore volume. The dashed line is provided as a visual guide (linear fit).

To investigate the sensitivity of the model in differentiating between coke of aromatic origin and noncyclic aliphatic coke deposited in CHA cages, we repeated the virtual coke deposition procedure with benzene and naphthalene molecules creating new theoretical aged CHA structures. Figure 7 presents the absolute deactivation predicted by our model and the same experimental data already shown in Figure 5. When aromatic molecules are used in place of heptane, the simulated values deviate from the experimental values mainly for the higher concentrations of carbon (above of 1.5 wt %). These calculations indicate that the volume of the CHA structure deactivated by aromatic molecules is lower than that deactivated by linear *n*-heptane molecules. This is a relevant result because demonstrates that the combination of experimental adsorption techniques with molecular simulation can identify the nature of the coke formed in the CHA cages.

**Figure 7:**
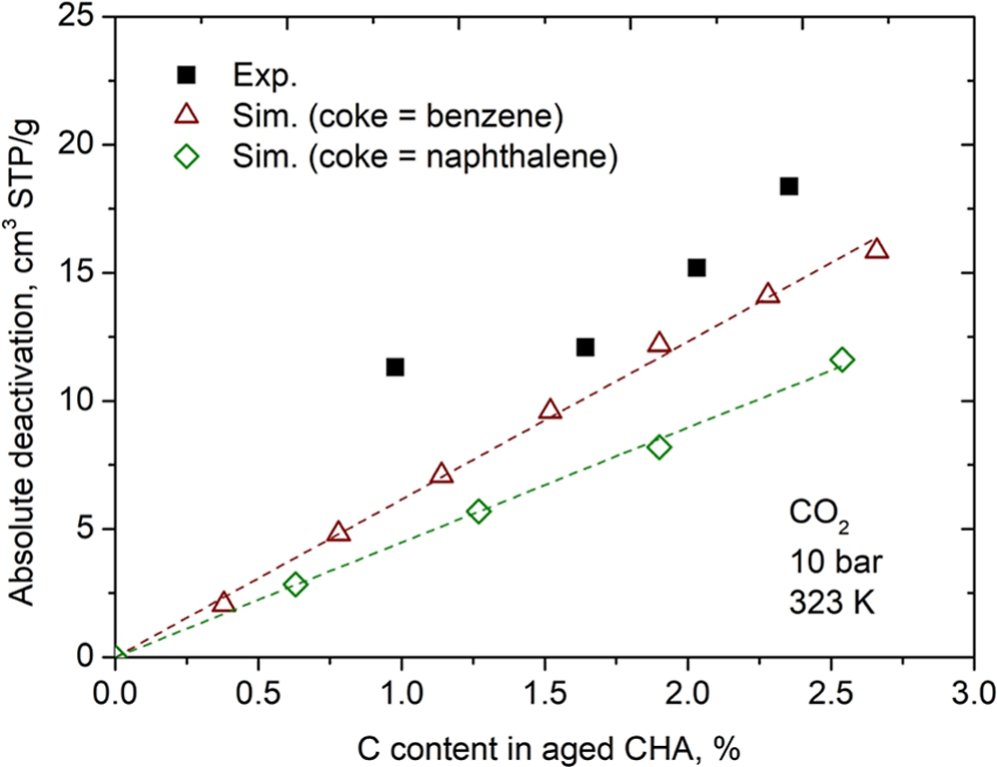
Absolute deactivation of CO_2_ at 323 K and 10 bar versus C content in aged CHA. Benzene or naphthalene were considered as coke in theoretical calculations. Data taken from Santiago et al.^11^ were used to carried out experimental calculations. The dashed lines are provided as a visual guide (linear fit).

Finally, we used the experimental isotherms of CO_2_ at 273 K on virgin CHA and aged CHA (only one experimental point with 2.35% C) from the study of Nascimento et al.^12^ to verify the performance of our model in estimating the deactivation of CHA measured under other temperature conditions. Figure 8 presents the simulated and experimental CO_2_ isotherms at 273 K for both fresh and aged samples. Once again, the simulated curves reproduce the experimental trends satisfactorily, considering the idealized nature of the virtual model relative to the real material, as previously discussed. This result indicates a good degree of transferability of the force field employed.

**Figure 8:**
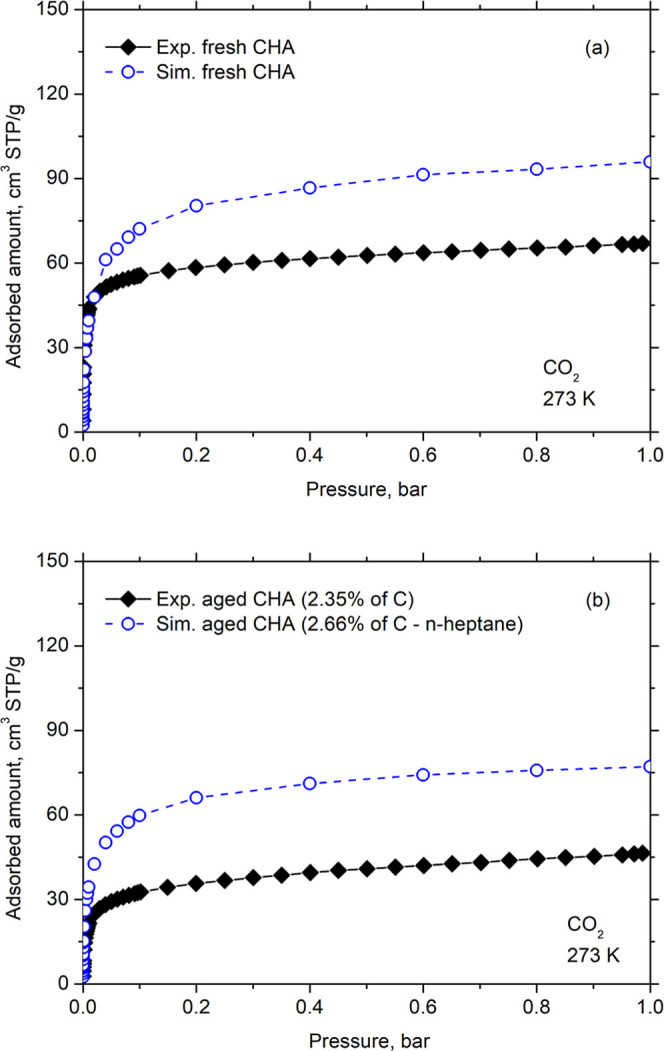
Adsorption isotherms of CO_2_ at 273 K in (a) fresh CHA and (b) aged CHA. Experimental data were taken from Nascimento et al.^12^

We repeated the procedure to calculate the absolute deactivation using simulated data at 273 K and compared with the experimental data available (Figure 9). The experimental value fits our deactivation prediction very well. The fact of being able to predict the deactivation in another condition of temperature and maximum pressure, indicates that the proposed model presents good transferability.

**Figure 9:**
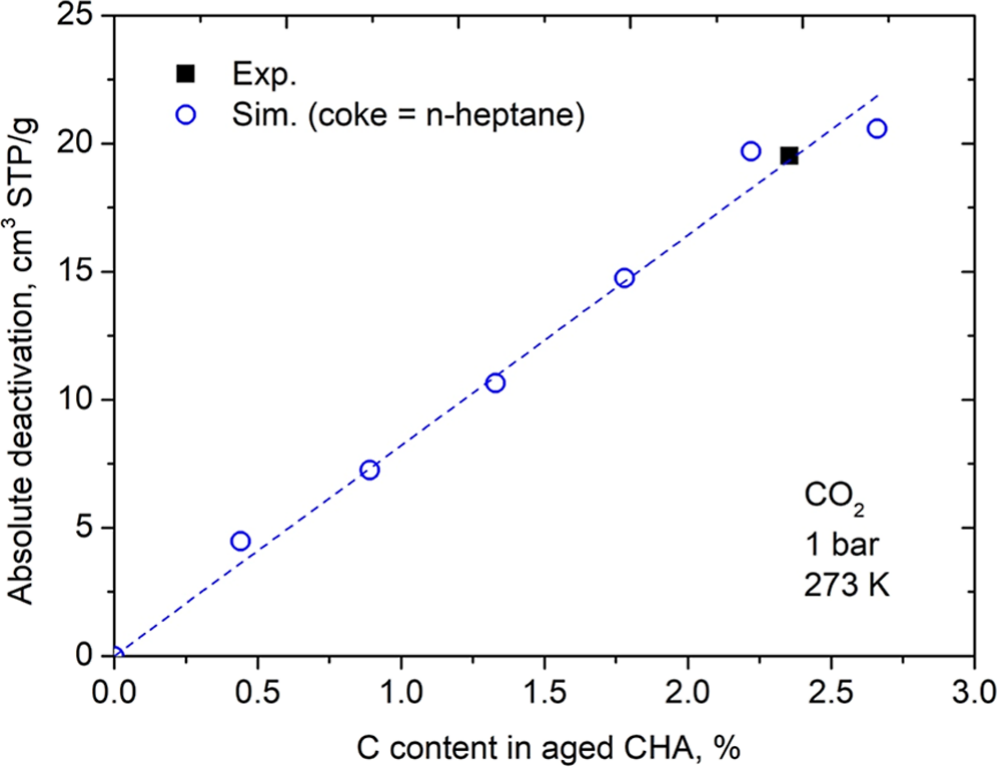
Absolute deactivation of CO_2_ at 273 K and 1 bar versus C content in aged CHA. *n*-Heptane was considered as coke in theoretical calculations. Data taken from Nascimento et al.^12^ were used to carried out experimental calculations. The dashed line is provided as a visual guide (linear fit).

### 3.5 Transferability

We observed that molecular simulation models for various sieves and coke-forming molecules can be developed to support the monitoring the deactivation of zeolite beds in industrial adsorption and catalysis units. To assess the transferability and the robustness of our methodology to different sieves, we conducted both experimental and virtual aging on LTA-type zeolite to evaluate deactivation. Additionally, we assessed deactivation in SAPO-34 using N_2_ adsorption data from the literature to test the effectiveness of the method with different probe molecules. This validation was strategically designed to test the performance of the approach across two key variables: the zeolite framework itself and the probe molecules used for diagnosis.

#### 3.5.1 Deactivation of LTA

We experimentally measured CO_2_ adsorption at 298 K and 1 bar in three different LTA samples: a pelletized sample and two pelletized aged samples at 523 and 573 K, following the procedure reported by Santiago et al.^11^ Additional information on the materials and experimental procedures is available in the work by Nascimento et al.^12^ Figure 10 shows the correlation between the measured absolute deactivation (in mmol/g) and the carbon content (%) in the aged samples. The sample aged at 523 K (LTA_523 K_) showed a carbon content of 2.2% and a deactivation of 1.02 mmol/g of CO_2_, while the sample aged at 573 K (LTA_573 K_) had a carbon content of 2.7% and a deactivation of 0.87 mmol/g. We observed that the LTA_573 K_ sample exhibited lower deactivation than LTA_523 K_ (0.87 vs 1.02 mmol/g), despite having a higher carbon content (2.7% vs 2.2%).

**Figure 10:**
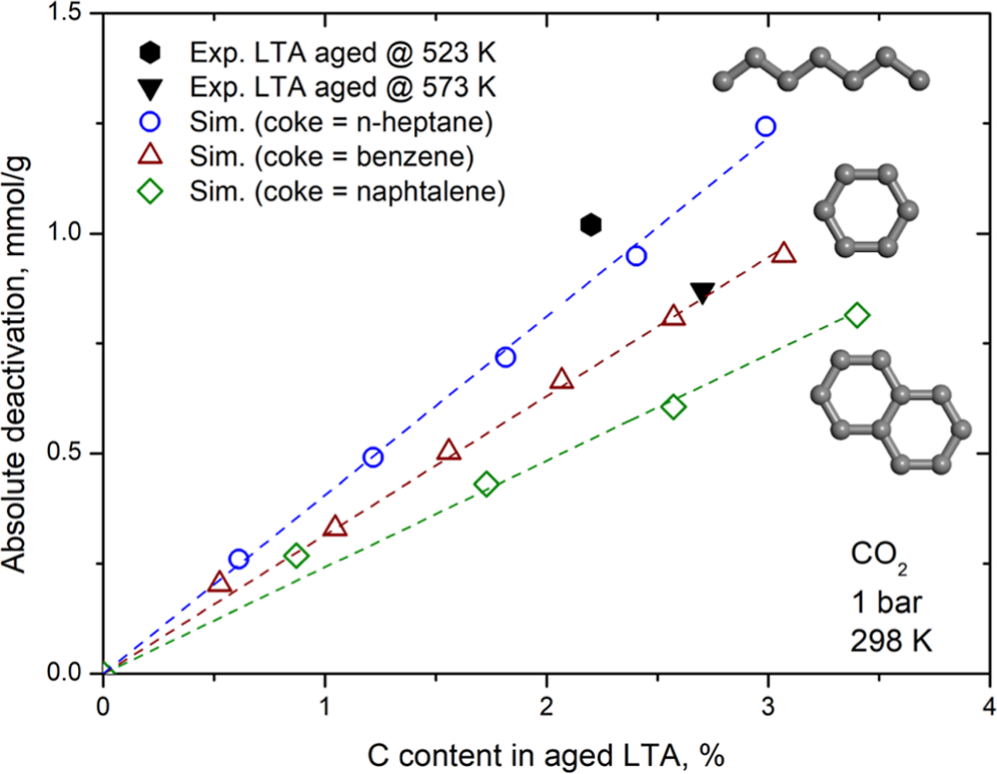
Absolute deactivation of CO_2_ at 298 K and 1 bar versus C content in aged LTA. *n*-Heptane, benzene, and naphthalene were considered as coke in theoretical calculations. The dashed lines are provided as a visual guide (linear fit). Molecular models of coke molecules are shown to illustrate their structural differences.

To investigate these results and the nature of the coke formed in each aged sample, we performed virtual aging on LTA using the same molecules we used previously for CHA. Up to 5, 6, and 4 molecules per unit cell of *n*-heptane, benzene, and naphthalene, respectively, were used in the procedure. We applied the force field recently validated by our group^24^ to calculate the amount of CO_2_ adsorbed at 298 K and 1 bar in the virgin LTA and the virtually aged structures. The theoretical predictions based on the simulated data are also presented in Figure 10.

Although the limited number of experimental points precludes a rigorous quantitative correlation between deactivation degree, coke loading, and coke nature, the results reveal a consistent qualitative trend. We see that the measured value for the LTA_523 K_ sample is like that estimated for the LTA virtually aged with *n*-heptane, while the measured deactivation for the LTA_573 K_ is close to that predicted for the LTA virtually aged with benzene. These results suggest that the aromatic coke formation was favored when the aging process reached 573 K. This aligns with what is reported in the literature, which states that increasing the reaction temperature favors the formation of aromatic and polyaromatic coke molecules.^36^ The underestimation of deactivation by the predictions for the LTA structures virtually aged with naphthalene, compared to the experimental results for both aged samples, indicates that polyaromatic coke did not form even in sample aged at 573 K.

#### 3.5.2 Deactivation of SAPO-34 Using N_2_ Adsorption Data

Gao et al.^7^ and Wang et al.^8^ investigated coke formation in SAPO-34 zeolite. Both studies used N_2_ isotherms at 77 K to monitor micropore volume throughout the deactivation process. We identified an excellent opportunity to test our methodology and predict the measured deactivation using a different probe gas (N_2_).

We constructed the SAPO-34 structure based on the CHA topology. From the all-silica structure,^37^ we randomly replaced Si atoms with Al and P, in accordance with Löwenstein’s rule, to obtain the molecular formula Si_0.085_Al_0.491_P_0.424_O_2_, as identified by Wang et al. We used the force field proposed by Talu and Myers^38^ to calculate the Helium Void Fraction (HVF) using the Widom particle insertion method^39^ and subsequently determined the accessible pore volume (total volume × HVF) in virgin SAPO-34. Averages were obtained from 100,000 configurational-biased insertions using the RASPA 2.0 code. We obtained an HVF of 0.40592 and an accessible pore volume of 0.2672 cm^3^/g. This value closely matches the micropore volume measured by Wang et al. through N_2_ adsorption at 77 K (0.251 cm^3^/g from the BET method). It is important to note that it is natural for the virtual structure to show a slightly higher pore volume than the experimental one, as it represents an ideal (perfect) structure, unlike the real crystal, which exhibit imperfections resulting from the synthesis process. From this virgin SAPO-34 structure, we performed the virtual aging procedure. As suggested by Gao et al., we used benzene and naphthalene molecules to represent coke up to 12 wt %. Up to 30 benzene molecules and 20 naphthalene molecules were used in the virtual aging. After the virtual aging, the aged structures had their HVF and accessible pore volumes calculated. Deactivation was calculated as the difference between the accessible volumes of the virgin structure and those of the aged ones.

Figure 11 presents the deactivation curves as a function of coke content from molecular simulation and those measured by Gao et al. and Wang et al. at 673 and 748 K, respectively. Once again, our methodology was able to predict deactivation satisfactorily. At low coke content (up to 3%), the deactivation curve of benzene closely matched the experimental data. At higher coke concentrations, the naphthalene deactivation curve better reproduced the experimental data. This result is consistent with what was observed by Gao et al., who found that at low coke content, coke mainly consists of (89% of molecules at 2.5 wt %) of monocyclic compounds (benzene class), and this percentage decreases as the reaction progresses due to the formation of bicyclic aromatics (naphthalene class) and polycyclic aromatics (in lower proportions). This result further reinforces the applicability and the transferability of the methodology developed in this study.

**Figure 11:**
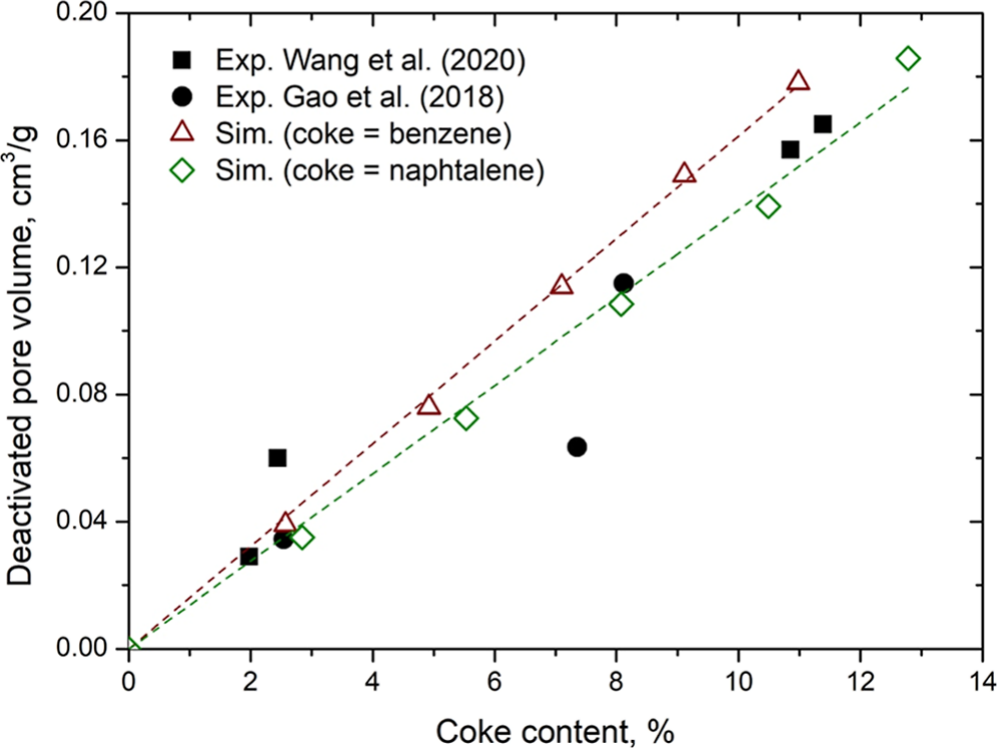
Deactivated pore volume versus coke content in aged SAPO-34. Benzene and naphthalene were considered as coke in theoretical calculations. Experimental data were obtained from N_2_ adsorption at 77 K by Gao et el.^7^ and Wang et al.^8^ The dashed lines are provided as a visual guide (linear fit).

### 3.6 Advantages and Limitations

Unlike conventional bulk techniques such as CHN or thermogravimetric analysis (TGA), this approach provides molecular-level insight into how coke formation reduces adsorption capacity. By linking atomic-scale structure to macroscopic performance, it enables the prediction of deactivation trends without requiring extensive experimental testing. This predictive capability is particularly valuable for applications involving gas mixtures, where experimental quantification of multicomponent adsorption is challenging due to the difficulty of accurately isolating individual adsorption contributions. The proposed approach allows for the estimation of mixture adsorption behavior and deactivation trends without the need for extensive multicomponent measurements, thereby offering a powerful complement to experimental techniques in process design and optimization.

A practical example of this advantage can be illustrated by Temperature Swing Adsorption (TSA) processes employed for natural gas dehydration.^40^ In these systems, zeolitic beds cyclically alternate between adsorption and regeneration steps, during which water is removed from the gas stream and subsequently desorbed by heating. Over repeated cycles, coke may form through hydrocarbon reactions, progressively blocking the micropores and active sites of the zeolite. This accumulation progressively diminishes the water uptake capacity and impairs regeneration efficiency. The proposed methodology can predict the gradual loss of water adsorption capacity before it severely impacts performance. As a result, bed lifetime can be extended, energy consumption during heating and cooling cycles reduced, and unplanned shutdowns due to premature desiccant failure avoided.

The main limitations stem from the molecular modeling foundations. Simulation accuracy depends on the reliability of the force fields and the representativeness of the simplified carbon-deposition models. Practical application may also be limited by the need for accurate structural and compositional input data and by uncertainties in describing the full complexity of coke species. Furthermore, the current framework does not capture the chemical kinetics of coke formation, focusing instead on the resulting structural and adsorption effects. Future work should integrate reactive simulations to enhance both the predictive power and industrial applicability of the methodology.

## 4 Conclusion

A molecular simulation model was developed to predict coke-induced deactivation in zeolite CHA. The model represents a typical natural zeolite containing a mixture of three cations (Ca^2+^, K^+^ and Na^+^) with a Si/Al ratio of 3. Validation against experimental CO_2_ adsorption isotherms at 323 and 273 K confirmed the accuracy of the structural model. The nature and the impact of coking were probed using *n*-heptane, benzene, and naphthalene as coke molecules. The integration of molecular simulation with experimental adsorption data demonstrated that this approach can both identify the nature of the coke and quantitatively correlate its concentration with the degree of adsorption deactivation. Furthermore, the model exhibited significant transferability, successfully predicting deactivation trends across different temperatures, in distinct zeolitic structures (LTA and SAPO-34), and with alternative probe molecules. This initial validation provides a strong foundation for future studies to explore an even broader portfolio of zeolites and coke molecules. This study introduces a novel strategy that integrates molecular simulation with experimental adsorption data to create a predictive tool, directly linking coke accumulation to quantifiable changes in adsorption performance. Unlike conventional techniques such as TGA or CHN analysis, this approach uniquely captures the spatial distribution of coke and its molecular-level impact on adsorbent functionality. This enhances its potential for critical applications including catalyst lifetime prediction, deactivation mechanism identification, and the design of optimized regeneration strategies, thereby serving as a complementary addition to existing characterization tools. Nonetheless, practical application may be constrained by the requirement for accurate structural and compositional input data and by uncertainties in representing the full complexity of coke species. Despite these challenges, this research fills a gap in the scarce literature on coke aging in adsorption. These findings encourage the use of adsorption as a complementary technique for investigating coking in catalysis, while pointing to future work aimed at refining the method for industrial implementation.
